# A Prospective Randomized Trial Comparing Effectiveness of Parasagittal and Midline Epidural Steroid Injection in Patients with Lumbar Canal Stenosis Pain

**DOI:** 10.5812/aapm-157791

**Published:** 2025-02-16

**Authors:** Masoud Hashemi, Faranak Behnaz, Payman Dadkhah, Ali Alizadeh Ojoor, Sina Hassannasab, Seyed Sam Mehdi Hosseininasab, Sogol Asgari

**Affiliations:** 1 Anesthesiology Research Center, Imam Hossein Hospital, Shahid Beheshti University of Medical Sciences, Tehran, Iran; 2 Shohada Tajrish Hospital, Shahid Beheshti University of Medical Sciences, Tehran, Iran; 3 Critical Care and Pain Medicine, Department of Anesthesiology, Anesthesia Research Center, Shahid Beheshti University of Medical Sciences, Tehran, Iran; 4 Shahid Beheshti University of Medical Sciences, Tehran, Iran; 5 Department of Anesthesiology, Anesthesiology Research Center, Loghman Hakim Hospital, Shahid Beheshti University of Medical Sciences, Tehran, Iran; 6 Anesthesiology Research Center, Shahid Beheshti University of Medical Sciences, Tehran, Iran

**Keywords:** Low Back Pain, Epidural Steroid Injections, Parasagittal Interlaminar, Spinal Canal Stenosis

## Abstract

**Background:**

Low back pain (LBP) due to lumbar spinal stenosis presents a significant clinical challenge. Epidural steroid injections (ESIs) are a common treatment option; however, the optimal injection route remains debated.

**Objectives:**

To compare the clinical outcomes of parasagittal interlaminar (PIL) versus midline interlaminar (MIL) ESI in patients with LBP attributed to lumbar spinal stenosis.

**Methods:**

This prospective, randomized study included patients with LBP and lumbar stenosis. Participants were randomly assigned to receive ESI via either the PIL or MIL route. Clinical outcomes, including pain intensity (measured by the Numeric Rating Scale [NRS]) and functional disability (assessed using the Modified Oswestry Disability Index [MODQ]), were evaluated at 1- and 3-months post-injection.

**Results:**

Analysis revealed a significant reduction in pain intensity (NRS) at 3 months post-injection in the PIL group compared to the MIL group (P = 0.014). Additionally, the PIL group demonstrated significantly lower patient satisfaction scores at 3 months (P = 0.033) and higher MODQ scores at 3 months (P = 0.002) compared to the MIL group. No significant differences were observed between groups at baseline or at the 1-month follow-up for any of the assessed outcomes.

**Conclusions:**

This study suggests potential differences in efficacy between parasagittal and midline interlaminar ESIs for lumbar stenosis pain. These findings underscore the need for further research to optimize treatment strategies and improve pain management for patients with this condition.

## 1. Background

Low back pain (LBP) can have diverse etiologies, including spinal stenosis, which itself may arise from vertebral bone irregularities and could be accompanied by intervertebral disc protrusion or herniation. Computed tomography (CT) and magnetic resonance imaging (MRI) are effective tools for evaluating spinal stenosis. Despite various grading methods for spinal stenosis, no single technique reliably predicts symptoms or ensures a favorable surgical outcome (1, 2). Renfrew's grading scheme, based on spinal imaging, utilizes the anterior-posterior (AP) diameter of the spinal canal. It compares the abnormal surface with the adjacent normal surface in the same patient to determine three degrees of peripheral canal stenosis: Mild, moderate, and severe stenosis (1). Various methods, including conservative management, epidural steroid injections (ESI), and surgical interventions, are employed in the treatment of LBP. If pain relief is insufficient after conservative management, corticosteroid epidural injections are indicated (3, 4). The theoretical basis for ESI lies in its ability to address the inflammatory reaction causing spinal nerve stimulation. Phospholipase A2, produced in abundance by a herniated disc, increases prostaglandin production, leading to inflammation and pain. Epidural steroid injections inhibits the synthesis or release of pro-inflammatory substances, alleviating the inflammatory response (5-8). The epidural space can be accessed through interlaminar (IL), caudal, or transforaminal (TF) routes (9). The ESI's effectiveness in pain relief hinges on administering the drug near the site of pathology, making the transforaminal TF technique potentially more efficacious. However, complications such as spinal cord injury and paraplegia have been reported with this technique due to Adamkiewicz artery embolization (10-12). The interlaminar (IL) route, specifically the midline interlaminar (MIL) and parasagittal interlaminar (PIL) paths, provides access to the posterior epidural space, avoiding spinous extravasation of the vertebrae (1, 13). Limited studies suggest that the PIL route may offer superior efficacy compared to the MIL route, possibly due to better abdominal drug spread, resulting in enhanced pain relief. The PIL route could potentially yield better results with fewer complications than the transforaminal TF route (13). 

## 2. Objectives

This study aims to compare the clinical outcomes of pain relief, functional improvement, and disability reduction at 1 and 3 months after epidural steroid administration using two methods: PIL and midline interlaminar.

## 3. Methods

### 3.1. Inclusion Criteria

Adults aged 18 to 65 years with clinical symptoms consistent with lumbar radiculopathy, including pain radiating to the lower extremities, were included. Compatibility of clinical symptoms with diagnostic imaging findings, such as MRI, confirmed the presence of conditions like spinal stenosis or herniated discs. Participants demonstrated willingness to provide informed consent for participation in the study, ensuring understanding of the procedure and potential risks. A medical evaluation confirmed suitability for lumbar ESI, including assessment of overall health and specific contraindications.

### 3.2. Exclusion Criteria

Contraindications to lumbar ESIs include active infections, bleeding disorders, or any condition that may increase the risk of complications. Allergy or hypersensitivity to the components of the injectate, such as steroids or local anesthetics, is also a contraindication. A history of previous adverse reactions to ESIs may indicate a higher risk for future procedures. The presence of significant psychiatric or medical comorbidities, such as severe depression or uncontrolled diabetes, could confound the interpretation of outcomes. Pregnancy or lactation may pose additional risks to both the mother and the fetus or infant. Inability to comply with study procedures or follow-up visits is essential for accurate data collection and outcome assessment. Participation in another clinical trial involving investigational drugs or interventions within the past 30 days should be avoided to prevent potential confounding effects on study outcomes. A history of lumbar surgery may alter the anatomy and affect the efficacy or safety of the injection procedure.

### 3.3. Randomization and Blinding

This clinical trial employed a double-blind randomization method to ensure the integrity of the study results. Neither the participants nor the researchers involved in the data collection and analysis were aware of the treatment allocation, thereby minimizing bias. Randomization was conducted using computerized algorithms to allocate participants into either group 1 or group 2 for each interlaminar route (parasagittal or midline). This method enhances the reliability of the findings by ensuring that any observed differences in outcomes can be attributed to the treatment rather than external factors. Additionally, close coordination between anesthesiologists and neurosurgeons was maintained throughout the study to ensure the selection of suitable patients and adherence to the study protocol.

### 3.4. Anesthesia Technique

After adhering to the specified inclusion criteria and obtaining informed consent from participants, a systematic process was followed. Initially, patients were randomly assigned to either group 1 or group 2 for each intravenous route. Subsequently, patients were positioned in a prone manner on the fluoroscopy table. Non-invasive measurements, including blood pressure, pulse oximetry, and electrocardiography, were conducted to monitor vital signs throughout the procedure. Intravenous sedation was achieved using a combination of midazolam (1 mg) and fentanyl (50 µg) administered incrementally to achieve the desired level of sedation while ensuring patient comfort. The lumbar vertebrae area was disinfected using either betadine or 10% povidone-iodine, followed by the application of a sterile facial covering to maintain a clean environment. Utilizing fluoroscopic imaging, the entry point for the needle was determined in the appropriate lumbar spine space. In the PIL group, the needle was introduced into the most lateral part of the epidural space at the target level and advanced in a posterior to anterior direction, maintaining the parasagittal orientation throughout the procedure. Local anesthesia was administered using 3-4 mL of 1% lidocaine to minimize discomfort during the injection. The resistance loss technique guided the advancement of a 3.5-inch, 19-gauge Tuohy needle to the epidural space. Anteroposterior and lateral fluoroscopy images were employed to guide the needle placement, utilizing both midline and parasagittal approaches. To enhance procedural safety, the lateral fluoroscopy view was used, ensuring that the final position of the needle tip was at the Ventral Interlaminar Line (VILL). Following the loss of resistance and negative aspiration for cerebrospinal fluid or blood, 2 mL of the radiocontrast agent (Omnipaque, 322 mOsm/kg water) was injected, and fluoroscopy was conducted to confirm the appropriate distribution of the contrast agent in the epidural space. Upon confirmation, a gradual injection of a 10 mL solution containing 0.1% ropivacaine and 40 mg triamcinolone was administered to provide both analgesic and anti-inflammatory effects. Patients were observed in bed for 30 minutes post-injection to monitor for any immediate adverse effects, and they were discharged if clinical symptoms remained stable without any complications. To assess outcomes, demographic characteristics and lumbar MRI findings were meticulously documented. The primary outcome involved evaluating the efficacy of lumbar midline interlaminar (IL) and lumbar parasagittal IL ESI based on scores from the Numeric Rating Scale (NRS) and the Modified Oswestry Disability Questionnaire (MODQ) at 1- and 3-month follow-ups. Pain intensity and functional status were quantified using NRS and MODQ scores before injection and at 1 and 3 months post-injection.

### 3.5. Statistical Analysis Method

In the statistical analysis of the study, descriptive statistics were employed to present continuous variables as means and standard deviations, while qualitative variables were expressed as counts and percentages. The comparison of quantitative variables between the two groups involved the use of Student's *t*-test, with the non-parametric Mann-Whitney test applied when necessary to account for non-normally distributed data. For qualitative variables, both the chi-square test and Fisher's exact test were utilized to compare differences between the two groups. To assess the trends of NRS and MODQ scores over time and to compare these trends between the two groups, a repeated measures analysis of variance (ANOVA) model was employed. This statistical approach allows for the examination of changes in variables measured repeatedly over time within groups and comparisons between different groups. All statistical analyses were conducted at a significance level of 5%, and the statistical software SPSS version 26 was utilized for data analysis, ensuring rigorous evaluation of the study outcomes.

## 4. Results

**Figure 1.**
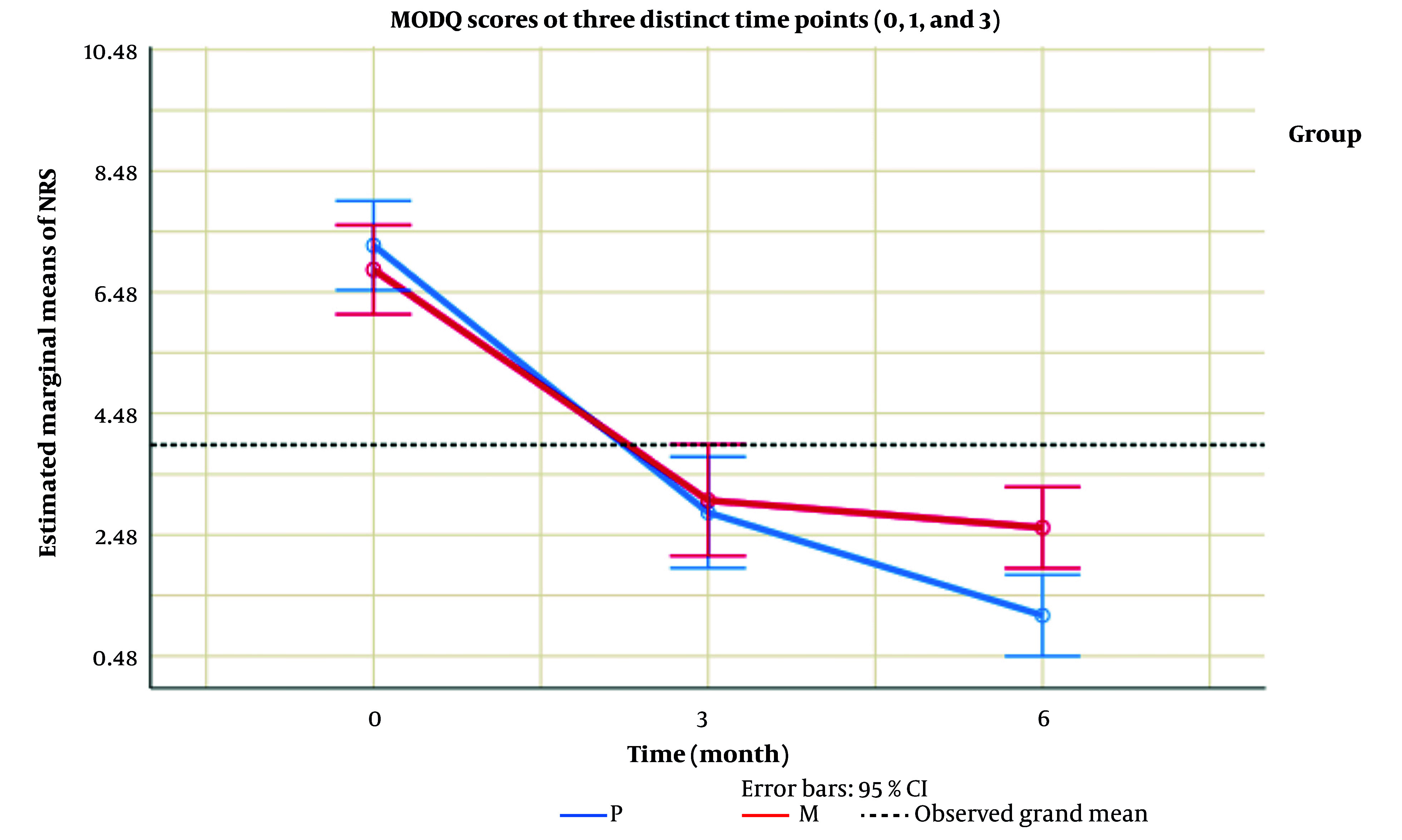
Numeric Rating Scale (NRS) scores at different time points

In the context of our investigation, the analysis presented in Table 1 unveiled no substantial distinctions in the age groups of the examined patients. Transitioning to Table 2 and Figure 1, where the NRS scores were scrutinized at various time points, no significant variations were discerned between the groups at times 0 and 1. However, a noteworthy finding emerged at time 3, indicating a statistically significant difference in the average NRS score between group M and group P (P = 0.014). This divergence was further accentuated by the visual representation in Table 2, showcasing a parallel trend in NRS scores over time with no significant overall differences between the two groups (P = 0.396).

**Table 1.** Age Groups of Patients ^a^

Variables	P (n = 20)	M (n = 20)	P-Value
**Gender**			0.752
Female	9 (45.0)	10 (50.0)	
Male	11 (55.0)	10 (50.0)	
**Age**	49.40 ± 7.53	49.05 ± 12.12	0.350

^a^ Values are expressed as No. (%) or mean ± SD.

**Table 2.** Numeric Rating Scale Scores at Different Time Points ^a^

Variable	P (n = 20)	M (n = 20)	P-Value
**NRS**			
0	7.25 ± 1.62	6.85 ± 1.63	0.429
1	2.85 ± 1.98	3.05 ± 2.06	0.773
3	1.15 ± 0.99	2.60 ± 1.85	0.014
**Total**	-	-	0.396 ^b^

Abbreviation: NRS, Numeric Rating Scale

^a^ Values are expressed as mean ± SD.

^b^ No significant variation between the groups were indicated.

**Figure 2.**
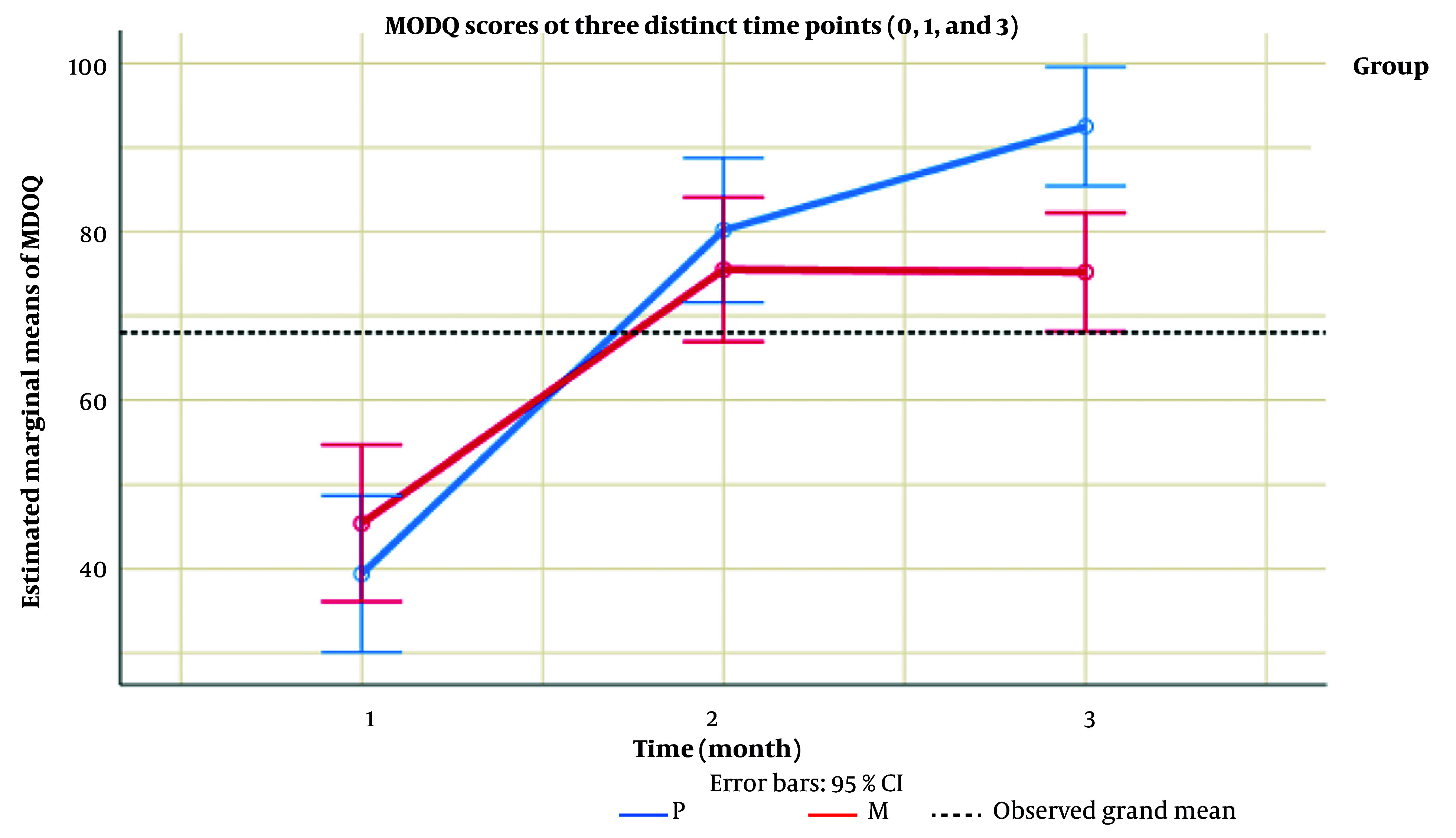
Modified Oswestry Disability Index (MODQ) scores at three distinct time points (0, 1 and 3)

Turning our attention to Table 3, the examination of R1 and R3 variables revealed a significant disparity in the average R3 scores between group P and group M (P = 0.033). Subsequently, Table 4 and Figure 2 delved into the MODQ scores at three distinct time points (0, 1, and 3), along with an overarching assessment of the trends over time. The outcomes highlighted a substantial difference in the average MODQ score at time 3, with group P exhibiting a significantly higher score than group M (P = 0.002). Notably, this contrast was absent at times 0 and 1. The longitudinal evaluation further indicated a lack of significant variation between the two groups over time (P = 0.316), a trend visually evident in Table 4. 

**Table 3.** Patient Satisfaction One Month and Three Months After Injection ^a^

Variables	P (n = 20)	M (n = 20)	P-Value
**R1**	4.25 ± 0.85	4.30 ± 0.98	0.738
**R3**	4.85 ± 0.37	4.10 ± 1.07	0.033

^a^ Values are expressed as mean ± SD.

**Table 4.** Modified Oswestry Disability Index Scores at Three Distinct Time Points (0, 1 and 3) ^a^

Variable	P (n = 20)	M (n = 20)	P-Value
**MODQ score (%)**			
0	39.40 ± 19.47	45.40 ± 21.52	0.183
1	80.20 ± 18.01	75.50 ± 19.78	0.398
3	92.50 ± 10.62	75.20 ± 19.35	0.002
**Total**	-	-	0.316 ^b^

Abbreviation: MODQ, Modified Oswestry Disability Index.

^a^ Values are expressed as mean ± SD.

^b^ No significant variation between the groups were indicated.

### 4.1. Summary of Findings

In summation, the comprehensive analysis of the results indicates distinct patterns and significant differences in certain variables, particularly in the context of NRS scores at time 3, patient satisfaction at R3, and MODQ scores at time 3. These findings contribute valuable insights into the efficacy of the PIL approach compared to the midline technique, highlighting the potential for improved pain relief and functional outcomes in patients undergoing ESI for chronic low back pain associated with spinal stenosis. The results suggest that while both approaches may offer initial relief, the parasagittal route may provide sustained benefits, enhancing patient satisfaction and reducing disability over time.

## 5. Discussion

Chronic pain remains a significant challenge in global healthcare, prompting extensive research aimed at developing innovative methods for pain relief and enhancing existing management strategies (14-19). Back pain, in particular, is a debilitating condition influenced by a variety of factors, including anatomical, psychological, and lifestyle components. Spinal canal stenosis, characterized by the narrowing of the spinal canal leading to compression of spinal cord and nerve structures, is a common cause of back pain (15, 20-22). This condition can result in a range of symptoms, including pain, muscular weakness, and sensory deficits (23, 24).

### 5.1. Rationale for Injection Route Selection

The route of injection in ESI procedures is critical, as it may greatly influence the distribution of the injected medication in the epidural space, thereby affecting treatment outcomes. This study aimed to compare the PIL route with the MIL route, with the hypothesis that the PIL route would allow for superior drug dispersion and potentially fewer complications than the MIL route. This hypothesis finds support from existing literature, which suggests that the PIL approach could afford advantages over the conventional MIL technique. Several analogous studies reinforce this rationale. For instance, another research conducted a comparative analysis evaluating the efficacy of ESIs via two different methodologies: Parasagittal and transforaminal approaches for back pain management. Their findings indicated that the lateral parasagittal approach demonstrated similar effectiveness to the transforaminal injection technique, leading to the suggestion that the lateral parasagittal method could be a viable alternative for back pain treatment.

### 5.2. Key Findings and Interpretation

The results of this study revealed several significant findings with important implications for clinical practice. While no significant differences were present in Numeric Rating Scale (NRS) scores between the PIL and MIL groups at baseline or at the 1-month follow-up, a statistically significant difference emerged at the 3-month assessment, with the PIL group experiencing greater pain alleviation. Furthermore, an analysis of the MODQ scores at the 3-month mark highlighted a significant reduction in disability for the PIL group compared to the MIL group. These outcomes suggest that the PIL route may provide enhanced long-term pain relief and functional improvement for patients suffering from low back pain associated with spinal stenosis. Further corroboration of this evidence comes from studies such as that conducted by Hashemi et al., who investigated the anatomical dispersion patterns of contrast media within the epidural space using fluoroscopic imaging (10). Their findings demonstrated that the parasagittal injection technique resulted in more extensive distribution of the injectate throughout the epidural space in the lumbar region compared to the midline approach, which was associated with greater clinical benefits and reduced disability linked to the parasagittal injection technique. Another relevant study by Ji Huntoon and Martin provided a comprehensive comparison of the efficacy of interlaminar parasagittal and transforaminal ESI techniques in patients with radicular neck pain (11). Both groups experienced a significant reduction in pain intensity at 1- and 3-month follow-ups, with the parasagittal approach yielding statistically significant advantages. The results pointed to the efficacy of the parasagittal injection technique in alleviating radicular neck pain compared to the transforaminal method. In the present study, while no statistically significant differences were detected between the outcomes of the PIL and Translaminar ESIs at initial assessments through the third-month follow-up, an evident contrast arose after the three-month mark. Patients receiving PIL injections reported higher satisfaction levels and lower pain than those who underwent Translaminar ESIs. These findings underscore the potential of the PIL technique to offer a more favorable long-term effect on both pain management and overall patient satisfaction. In summary, the evidence presented substantiates the hypothesis that the parasagittal route in lumbar ESIs could provide improved outcomes in pain relief and functional capacity. This study’s findings contribute valuable insights to the ongoing dialogue regarding optimal injection techniques for chronic back pain management, advocating for a shift towards more effective treatment modalities tailored to patient needs (13).

### 5.3. Limitations and Future Directions

Recognizing the constraints inherent in the study is imperative, encompassing its prospective nature, modest sample size, and restricted follow-up timeframe. The sample size is small and should be considered a study limitation. Subsequent research endeavors should endeavor to mitigate these limitations by executing expansive, multicenter investigations with extended monitoring intervals to corroborate the applicability and sustainability of the noted findings. Furthermore, delving into additional variables that potentially influence treatment efficacy, such as patient demographics, comorbid conditions, and procedural nuances, holds promise for yielding additional perspectives on refining therapeutic strategies for addressing low back pain linked to spinal stenosis.

### 5.4. Conclusions

In conclusion, this study provides a comprehensive evaluation of the efficacy of parasagittal versus midline interlaminar ESIs in patients suffering from low back pain due to spinal stenosis. The findings underscore the importance of individualized treatment strategies in pain management, highlighting the potential differences in effectiveness between these two injection techniques. Specifically, the data suggest that the parasagittal approach may offer superior outcomes in terms of sustained pain relief and improved functional status compared to the midline approach. These results not only point to the need for further research to validate and refine pain management protocols but also emphasize the necessity of tailoring treatment methods to meet the unique needs of each patient. Additional studies are warranted to explore the long-term effects and safety profiles of these injection techniques, as well as to identify the most appropriate patient populations for each method. Ultimately, advancing the understanding of these therapeutic modalities can significantly enhance patient outcomes and contribute to the development of more effective interventions for individuals experiencing low back pain associated with spinal stenosis.

## Data Availability

The dataset presented in the study is available on request from the corresponding author during submission or after publication.

## Clinical Trial Registration Code:

IRCT20131124015515N11.
